# GlueFinder: A Data-Driven Framework for the Rational Discovery of Molecular Glues

**DOI:** 10.1021/acs.jcim.5c03232

**Published:** 2026-03-03

**Authors:** Jeffrey Skolnick, Bharath Srinivasan, Hongyi Zhou

**Affiliations:** † Center for the Study of Systems Biology, 1372 Georgia Institute of Technology, 950 Atlantic Dr NW, Atlanta, Georgia 30332, United States; ‡ School of Pharmacy and Life Sciences, 1018 Robert Gordon University, Garthdee House, Garthdee Rd, Aberdeen AB10 7AQ, U.K.; § Department of Chemistry, Stony Brook University, Stony Brook, New York 11794-3400, United States; ∥ Center for the Advanced Study of Drug Action, Stony Brook University, Stony Brook, New York 11794-3400, United States; ⊥ Cancer Research Horizons, Cancer Research U.K, 2 Redman Place, London E20 1JQ, U.K.

## Abstract

Molecular glues drive targeted protein degradation by stabilizing ternary complexes between proteins of interest and E3 ubiquitin ligases, but their rational design has lagged due to a limited understanding of the rules for interface recognition and an overreliance on a few ligases (e.g., VHL or Cereblon). We introduce GlueFinder, a systematic, unbiased platform that leverages structural bioinformatics to mine the Protein Data Bank for ligand-binding pockets adjacent to the protein interface which are ligandable sites that can nucleate glue-mediated complex formation. After validating its performance on a benchmark of experimentally solved dimeric structures with known and predicted glues, we applied GlueFinder to three therapeutically important targets, EGFR, HER2, and KRAS, and predicted candidate glues that recruit 24, 111, and 148 distinct E3 ligases to these targets, respectively. We further demonstrate that GlueFinder can promote the formation of non-native EGFR complexes with a variety of diverse proteins, possibly enabling ternary assemblies that would not form on their own. These results establish a general, computation-guided experimental prioritization strategy for molecular glue discovery that decouples design from legacy degrader scaffolds and specific ligase dependencies, expands the usable E3 ligase repertoire, and enables rational targeting of interfacial binding pockets.

## Graphical abstract

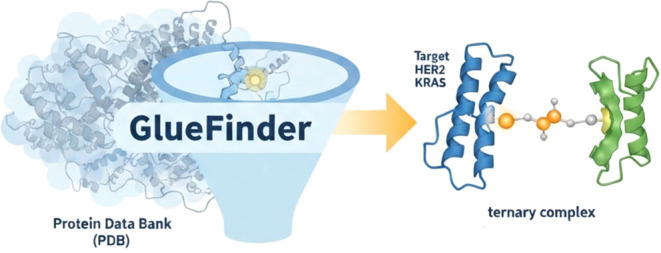

## Introduction

Molecular glues are an emerging class of small molecules that act by stabilizing protein–protein interactions (PPIs), enabling novel therapeutic mechanisms that diverge significantly from conventional small-molecule inhibitors.^1^ Unlike traditional drugs, which typically inhibit the activity of a single target protein by occupying active or allosteric sites, as shown in Figure 1, molecular glues function by *inducing or stabilizing* interactions between two proteins, that do not natively interact, often between a target protein and a cellular effector, thereby reprogramming protein function or fate. This mechanism of action also sets molecular glues apart from proteolysis-targeting chimeras (PROTACs), which rely on bifunctional molecules to physically tether a target protein to an E3 ubiquitin ligase.^2^ While PROTACs require defined binding motifs for both the target and the ligase, molecular glues operate through subtle surface complementarity and induced proximity, often modifying protein–protein interactions that are otherwise transient or nonexistent (see Figure 1).

**Figure 1:**
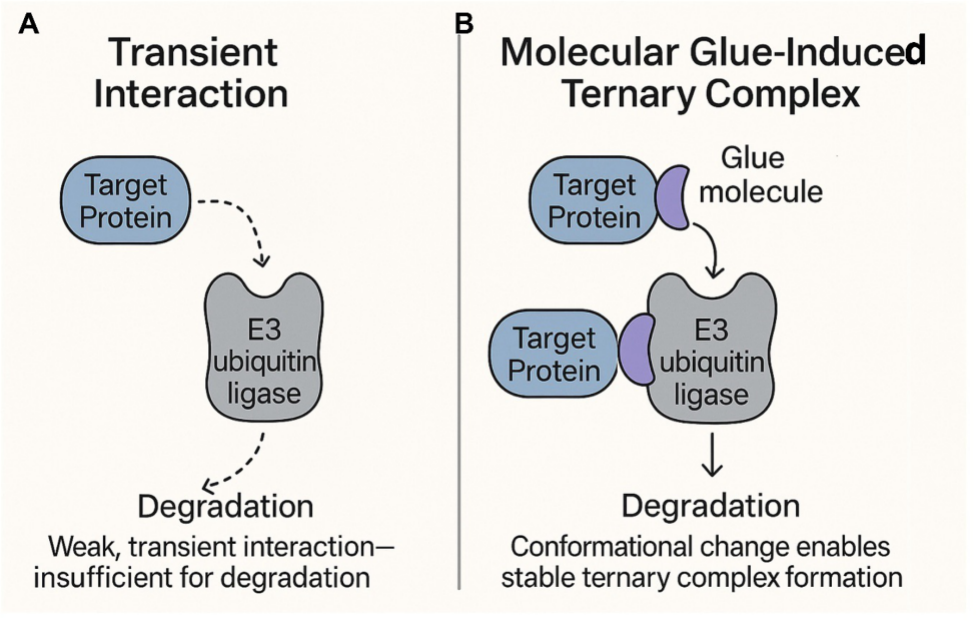
**A**. Schematic representation of mechanism of action for molecular glues. In the cellular milieu, protein–protein interactions are often transient. **B.** Molecular glues, as emphasized by the example of molecular glue degraders shown here, stabilize these interactions and make them constitutive, resulting in rewiring the cellular metabolic machinery and controls.

Molecular glues offer several compelling advantages. Their small size facilitates better cell permeability, blood-brain barrier (BBB) penetrance and favorable pharmacokinetic properties compared to larger bifunctional molecules like PROTACs.^1,3^ They can also access previously “undruggable” proteins that lack suitable binding pockets for traditional inhibitors or induce protein–protein complexes when the complex would be unstable in the absence of the molecular glue. Moreover, by leveraging endogenous degradation or signaling pathways, molecular glues can exert catalytic effects, modulating protein function even at substoichiometric doses.^4^ However, their development presents significant challenges. Most molecular glues have been discovered serendipitously, often through phenotypic screening rather than rational design.^3^ The precise molecular determinants that govern glue-induced ternary complex formation and functional outcomes remain incompletely understood, limiting the ability to generalize from known scaffolds or targets.

The therapeutic promise of molecular glues is becoming increasingly evident, particularly in oncology, where they have shown effectiveness in degrading oncogenic transcription factors and other difficult-to-target proteins.^2^ For instance, thalidomide and its analogues (IMiDs) have demonstrated the power of molecular glues to modulate substrate recognition by E3 ligases such as Cereblon (CRBN), leading to targeted degradation of neosubstrates like IKZF1/3.^5^ Beyond cancer, molecular glues are being explored for neurodegenerative diseases, immunological disorders, and viral infections, expanding their reach as a broad therapeutic modality.^1,6^

There have been several prior approaches to predict molecular glues. For example, Mayor-Ruiz et al.^7^ developed a chemical screening approach in hypo-neddylated cells followed by target deconvolution with the goal of finding E3 ligase molecular glues. Work by Weiss et al.^8^ focuses on the prediction of the relative degradation of cereblon E3 ligase modulators. Another study^9^ identified a molecular handle that led to improved CD4 degraders. More recently, Petzold et al.^10^ presented a general framework for identifying and exploiting interface-adjacent ligand-binding pockets in protein–protein complexes as reusable scaffolds for molecular glue design, beginning with systematic analysis of solved structures and validation against known ligand-bound dimers. Benchmarking and subsequent applications to human dimers and AlphaFold3-predicted weak complexes, particularly E3 ligases with targets such as EGFR and KRAS, support the hypothesis that the space of interface pockets is finite and transferable, enabling systematic prediction of small molecules that stabilize or induce protein–protein interactions. This idea provides further support of our much earlier analysis^11,12^ that builds on even earlier studies that showed that the space of ligand binding pockets is rather small and finite.^13^ Here, it was applied to a specific class of molecules and pockets but a general algorithm was not described. A very useful review describing the state of the art of molecular glues for E3-ligases is found in ref^14^.

A notable subclass of these molecules, molecular glue degraders, function by enhancing or enforcing interactions between E3 ubiquitin ligases and target proteins of interest (POI), leading to ubiquitin-mediated proteasomal degradation (Figure 1). These degraders essentially transform weak or transient PPIs into stable complexes, redirecting the cellular degradation machinery to specific targets.^15^ By reprogramming the substrate specificity of E3 ligases, they offer a powerful strategy for selectively depleting disease-causing proteins, including those lacking enzymatic activity or conventional drug-binding sites. A very prominent class of molecular glue degraders are the immunomodulatory imide drugs (IMiDs) such as thalidomide and its derivatives, lenalidomide (Revlimid) and pomalidomide (Pomalyst)^5^ (Figure 2). These compounds bind to the Cereblon (CRBN) protein, which is part of a larger ubiquitin ligase complex. This binding creates a new interaction interface which effectively as a “glue” to recruit specific target proteins, such as the transcription factors IKZF1 and IKZF3, to the CRBN-E3 ligase complex.

**Figure 2:**
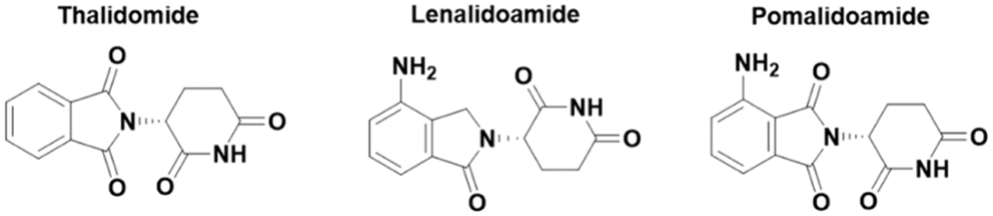
Examples of the IMiD class of molecular glue degraders thalidomide and its derivatives, lenalidomide (Revlimid) and pomalidomide (Pomalyst). These recruit CRBN E3 ligase to degrade the protein targets of interest.

Despite their potential, rational discovery and design of molecular glues remain an unsolved problem, largely due to the complexity of PPI surfaces, the dynamic nature of ternary complex formation, the absence of predictive computational or statistical frameworks and the traditional emphasis on two E3 ligases, i.e., VHL and Cereblon.^6^ Current approaches heavily rely on trial-and-error or serendipitous screening, which is both inefficient and difficult to scale. A robust, unbiased computational platform capable of systematically integrating structural, biophysical, and chemical data to model and predict molecular glue interactions would represent a transformative step forward. Such a platform could systematically identify new glueable targets, prioritize compound libraries, and optimize lead compounds with greater precision, thus accelerating the development of molecular glues across therapeutic areas.

Our work establishes such an unbiased computation guided approach for molecular glue discovery, decoupling design from known degrader scaffolds and specific ligase dependencies. By expanding the accessible E3 ligase landscape beyond CRBN and VHL and enabling rational targeting of interface adjacent binding pockets for an arbitrary collection of possible protein partners, our platform significantly broadens the scope and precision of targeted protein degradation, thus offering a transformative step toward systematic and mechanism-driven molecular glue development for diverse therapeutic applications.

This work presents a general framework for discovering and exploiting interface-adjacent ligand-binding pockets (IAPs) as scaffolds for molecular glue design. We begin by systematically analyzing experimentally solved protein structures to characterize the geometric and chemical nature of ligands that occupy pockets near protein–protein interfaces, establishing that these cavities are recurrent, stereochemically constrained, and therefore transferable across systems. A canonical example—the thalidomide–cereblon–CUL4 complex—demonstrates that clinically successful molecular glues frequently bind preexisting interface-adjacent pockets rather than creating entirely new contacts. We then validate the predictive methodology through postdiction benchmarks on known protein dimers, showing strong precision, recall, and pose recovery, and confirm the robustness and transferability of the scoring metrics. Having established reliability, we extend the approach to human dimers lacking annotated interface ligands and subsequently to weak or transient complexes modeled with AlphaFold3-Complex, focusing on E3 ligases and glues involving oncogenic targets such as EGFR, HER2, and KRAS and a plethora of other proteins. Across these analyses, the results support the hypothesis that the space of protein and interface pockets is finite and reusable, enabling systematic prediction of small-molecule glues that stabilize or induce protein–protein interactions and providing a broadly applicable strategy for rational molecular glue discovery.

## Results

### Analysis of Interface Adjacent Pocket Binding Ligands as Seed Scaffolds for MGD Design

As a first step, we explored the nature of ligands that bind to interface adjacent pockets in native protein dimers deposited in the Protein Data Bank (PDB). While these ligands are not molecular glues in the strict sense—that is, they do not induce novel interactions between proteins that otherwise would not associate—they nonetheless play an analogous physiological role by stabilizing protein–protein interactions.

Despite the naïve expectation that these are just crystallization artifacts, specific lipids play important roles in the modulation of protein structure and function.^20^

In addition, we found that six metal ions, Cd, Cu, Fe, Mg, Ni and Zn, are IAP binding ligands. Notably, some, including Zn, Fe and Cd, clearly stabilize protein structures and often contribute to enzymatic function.^21^ This information on interfacial metal ions could be leveraged to elucidate the principles of molecular glue design. Protein-metal ion binding affinity is dictated by the Irving-Williams (IW) series (Mn^2+^ < Fe^2+^ < Co^2+^ < Ni^2+^ < Cu^2+^ > Zn^2+^) with most metalloenzymes complexing with Zn^2+^ on one end of the spectrum and most metal-dependent enzymes binding to Mn^2+^/Mg^2+^ on the other end of the spectrum.^22^ Cognate metal speciation is a function of protein dynamics, nonequilibrium kinetic barriers and natural selection rules that dictate that the tightest binding metals are those that are least available.^23^ In fact, this know-how has been leveraged to design appropriate metal ion selectivity in protein design.^15^ A systematic analysis of interface adjacent metal ions suggests the following: From most to least frequent are Fe(10) > Cd(9) > Cu(7) and Mg(7) > Ni(4) > Zn(3), where the parentheses indicates the number of examples found in the PDB. We note that while there are too few examples to tease apart the ground rules for interfacial metal ion recognition, they are roughly in the same inverse order as the IW series.

In total, 1665 ligands were identified as IAP binding small molecules with the potential to act as molecular glues. The most common is glycerol. Lest one think that the presence of glycerol is merely a crystallization artifact, glycerol plays an important role in protein stabilization as a cosolvent effect^17^ but since it is found in IAPs the implication is that it may play a specific role in protein–protein interaction stabilization. In fact, triols are versatile fragments in fragment-based hit optimization exercises, offering multiple hydrogen bonding sites for weak but specific protein interactions. They are an aid in fragment linking/growing strategies, and help map binding pockets, especially for “undruggable” targets, by revealing regions with favorable polarity and geometry, that then results in potent leads through structure-guided optimization.^18^ Furthermore, the specific interaction of glycerol in protein pockets has been shown to orthosterically displace cognate binding ligands. The next most common ligand is ethyl 4-azanyl-3-bromanyl-benzoate. Another interesting molecule is pyroxidal-5′-phosphate, the active form of vitamin B6.^19^ Small organic molecules such as acetate and citric acid are observed at interfaces, along with a diverse collection of phospholipids that preferentially localize adjacent to protein dimeric interfaces.

Taken together, these findings highlight the idea that small molecules which bind interface-adjacent pockets represent a mechanistically diverse group of ligands that might potentially help stabilize multimeric protein assemblies and act as molecular glues. Furthermore, this analysis highlights the potential of these interface adjacent molecules to serve as a library of fragments or potential cores that would form the starting point for development of molecular glues by bioisosteric replacements and scaffold-hopping among the protein pairs. These changes would potentially serve to introduce favorable changes in molecular shape and size, electronic properties, p*K*_a_, and polarizability that will potentially facilitate tighter interactions across the protein pairs. They could transform transient interactions into permanent ones with reduced *k*_*off*_ for the bimolecular complexes. A natural question is what fraction of all PDB ligands function in this capacity? We have found that IAP binding ligands account for roughly 12% of all bound types of ligands in solved protein dimers. They span a wide spectrum, ranging from large cofactors such as hemes, to less physiologically relevant entities such as sulfate ions that may or may not mediate meaningful interactions.^16^

There are a total of 850 clusters when IAP ligands are clustered with a Tanimoto coefficient threshold of 0.8 using Daylight fingerprints.^24^ The top 19 clusters contain 10 or more members. The cluster centroid ligands for the top ten clusters along with the number of members per cluster are shown in Figure 3. These ligand clusters encompass a chemically diverse set of entities, ranging from highly charged groups to neutral scaffolds. A prominent feature is the prevalence of oxygen-rich functionalities which contribute polarity and hydrogen-bonding potential. This also makes sense in that these ligands are partly exposed to the cytosol when they bind. Excluding polyethylene glycols—the fourth largest cluster—many display drug-like characteristics. In addition, a significant subset is lipid-like with long hydrocarbon tails. In the Protein Data Bank, hydrocarbons frequently occupy interface-adjacent pockets as well as within protein–protein interfaces, exploiting their conformational flexibility to adapt to local stereochemistry.^11^ A substantial fraction of these ligand clusters represents monovalent small molecules with molecular weights of less than 500 Da, characteristic features of a molecular glue. Prominent examples include fluorotyrosine-like, (YOF) amino pyridoxal phosphate-like (PMP) and hexyl beta-d-glucopyranoside-like (JZR) molecules; see Figure 3. The complete ligand set is provided in the Supporting Information.

**Figure 3:**
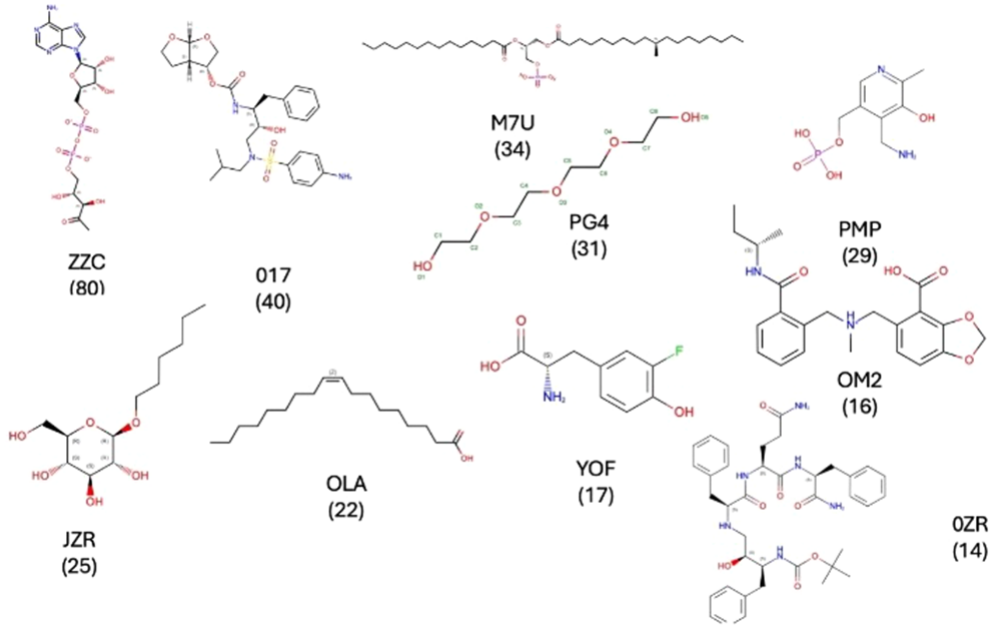
Cluster centroids of the top 10 clusters of interface adjacent ligands found in the PDB: ZZC ([(2r,3s,4r,5r)-5-(6-aminopurin-9-yl)-3,4-dihydroxy-oxolan-2-yl]­methyl [[(2r,3r)-2,3-dihydroxy-4-oxo-pentoxy]-oxido-phosphoryl] phosphate); 017 (3R,3S,6R)-hexahydrofuro­[2,3-*b*]­furan-3-yl­(1s,2r)-3-[[(4-aminophenyl) sulfonyl] (isobutyl)­amino]-1-benzyl-2-hydroxypropylcarbamate); M7U (2R)-2-(hexadecanoyloxy)-3­([(10r)-10- methyloctadecanoyl]­oxy}­propyl phosphate; PG4 (tetraethylene glycol); PMP (4’-deoxy-4’-aminopyridoxal-5′-phosphate); JZR (hexyl d-glucopyranoside); OLA (oleic acid); YOF (3-fluorotyrosine); OM2 ((R)-[2-[[(2S)-butan-2-yl]­carbamoyl]­phenyl]­methyl-[(4-carboxy-1,3-benzodioxol-5-yl)­methyl]-methyl-azanium); and OZR (boc-ps0-phe-gln-phe-NH2).

### Validation of GlueFinder’s Ability to Predict IAP Ligands in the Native Protein Dimers

As a preliminary step toward validating the utility of GlueFinder for predicting ligands that bind to pockets adjacent to protein–protein interfaces, we constructed a benchmark set of 238 solved human protein dimer structures from the PDB (see Materials and Methods for details). Since these dimers already have experimentally determined structures, this test evaluates the algorithm’s ability to predict and quantify GlueFinder’s precision and recall and binding pose prediction quality. As summarized in Table 1, we considered two scenarios. In the first, cases were restricted to those whose APoc^25^ (our pocket structure alignment algorithm) assigned p-value of the template ligand–target pocket structural similarity score was less than 0.05. Within this set, the most stringent condition required template ligands to reside in proteins with no more than 30% sequence identity to their template protein with either monomer that is found in the target dimer. Under these conditions, the recall was ∼24%, with an average center-of-mass root-mean-square-deviation, RMSD, of 3.01 Å. Relaxing the sequence identity threshold to 40%, beyond which results were largely insensitive, increased coverage to slightly under one-third of the database, with an average RMSD of 2.82 Å. In the second analysis, we applied a more stringent criterion of pocket similarity with a p-value ≤ 0.01, again restricting template ligands to ≤30% sequence identity. Now, ∼7.4% of ligands were correctly matched, with an average RMSD of 2.57 Å. Increasing the sequence identity threshold to 40% yielded an average RMSD ≤ 2.39 Å, and the coverage increased to 12%. Although the overall coverage was lower than in the more permissive case (i.e., when the p-value <0.05), the increased accuracy was modest, suggesting that an APoc p-value cutoff of 0.05 provides a robust balance of coverage and precision.

**Table 1:** Analysis of the Ability to Predict Native Glue-like Ligands in the 238 Protein Dimer Benchmark Set

Template sequence identity cutoff	Fraction of native ligands predicted	Average predicted precision per native ligand	Average RMSD of the predicted native ligand’s center of mass (Å)
**p-value ≤ 0.05**			
30%	0.24 (58/238)	0.16	3.01
40%	0.32(77/238)	0.19	2.82
50%	0.32(77/238)	0.19	2.81
70%	0.33 (79/238)	0.19	2.74
**p-value ≤ 0.01**			
30%	0.074 (20/238)	0.27	2.57
40%	0.12(41/238)	0.28	2.39
50%	0.17(41/238)	0.26	2.39
70%	0.18(42/238)	0.28	2.34

As a concrete illustration of the accuracy of the approach, as shown in Figure 4, with a template sequence identity cutoff of 30% we successfully predicted that thalidomide (EF2) binds to the complex of CRBN to SALL4,^26^ in agreement with experiment (PDB ID: 7BQU). The pose of EF2 is remarkably well predicted. This is an interesting case as the thalidomide pose is provided by the 5amk which is the monomeric structure of Cereblon. Indeed, one mechanism of ligand selection is to look for monomeric proteins that have a bound ligand one end of which sticks out into the solvent. The solvent exposed piece can then interact with the appropriate pocket to generate binding of the two protein partners. This is exactly what happens with thalidomide.

**Figure 4:**
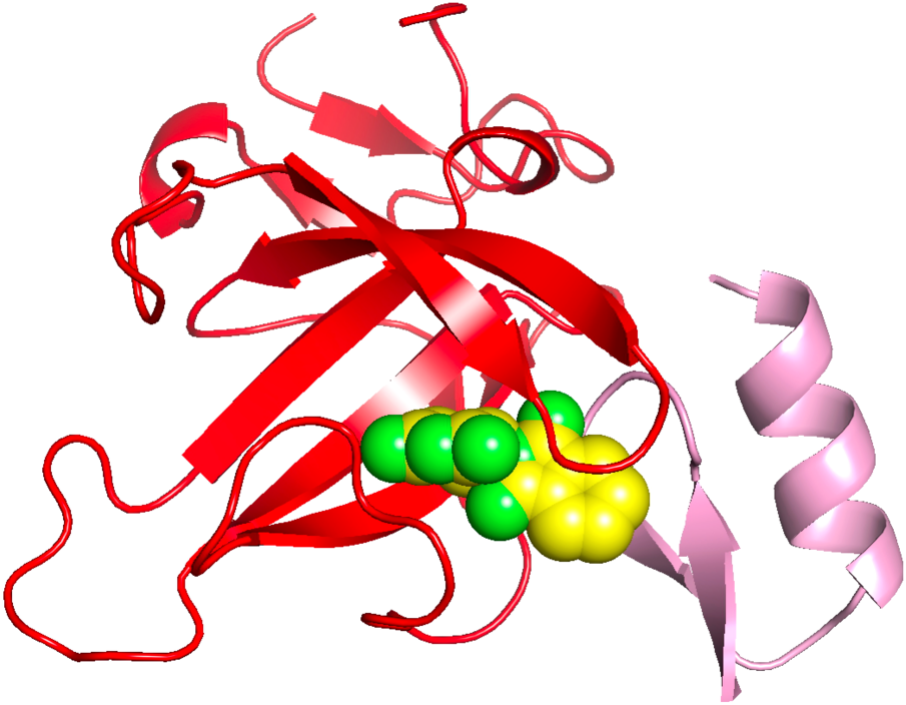
Predicted binding pose of thalidomide (green) compared to the experimental pose in the interface adjacent region between Cereblon (red) and SALL4 in 7BQU.^26^

For pomalidomide binding to CRBN and IKAROS, the CRBN domain in which pomalidomide is bound has a RMSD of 1.12 Å to the monomeric structure found in 5ank; however, it forms an interface adjacent ligand with IKAROS. In a number of other cases, the structure alignment of monomeric CRBN (PDB ID: 8rq8A^27^) to its structure found in 4Ci1B,^28^ 8oizB,^29^ 8g66B,^30^ 5fqdB,^31^ 5hxbC,^32^ 6h0gB,^33^ 8u16A,^34^ and 9tnpB^35^ dimers all have RMSDs below 2.1Å. 8rq8A (a monomer) lacks an inserted helical hairpin that interacts with the partner protein. The structure of 8tnp is typical of that where CBRN interacts with DNA damage protein 1 and pomalidomide is an IAP binding ligand (see Figure S1 and the superposition onto the monomeric 8rq8A and S1B Figure S2). The structural alignment of CRBN in 8rq8A and 6hf0B is 3.44Å (see Figure S2) where the pomalidomide binding domain opens and detaches from the remainder of the protein to interact with IKAROS. However, pomalidomide is still an IAP binding ligand. Thus, based on the above results, these findings establish a practical framework for employing GlueFinder in virtual ligand screening, which we will expand upon next.

### Application to Human Dimers without Known Bound Interface Adjacent Ligands

We next applied GlueFinder to a benchmark set of 2,682 human protein dimers from the PDB to evaluate its ability to predict IAP ligands for a diverse collection of human proteins, the “Dimer” set. The goals of this analysis were 2-fold: (i) to determine the fraction of dimers that are predicted to contain at least one ligand that putatively binds adjacent to the protein–protein interface, and (ii) to assess the predictive performance of the method in terms of precision and recall. As shown in Table 2, GlueFinder identified IAP ligands for 78% of native dimers, indicating that intermolecular glue candidates are potentially widespread across human protein complexes. In the absence of a target-template sequence identity cutoff, at a predicted binding precision threshold of 0.15, 2,089 proteins have a predicted average binding precision of 0.18, with the best average binding precision of 0.24. A given binding precision of 0.18 means that 18% of the ligands are predicted to bind with a 10 μM or better binding affinity based on experimental validation.^12,36^ The black curve in Figure 5 shows the cumulative histogram of the predicted binding precision. At a template sequence identity cutoff of 30%, and a minimum predicted precision of 0.15, 2,083 dimers are predicted to have interface adjacent binding ligands (see Supporting Information). The average binding precision and fraction of known interface adjacent binding ligands is virtually the same as when a permissive sequence identity cutoff is used. The red curve in Figure 5 shows the plot of the cumulative fraction of predicted ligands versus the predicted binding precision. While the average precision is 18%, roughly 13% of the ligands have a predicted binding precision above 20%. This result suggests that a substantial fraction is likely to represent true positives, with the best having a predicted binding precision of 0.59, thereby supporting the utility of GlueFinder in prioritizing small molecules for subsequent experimental validation of their glueing potential. Interestingly, the cumulative fraction of cases with a predicted binding precision is very insensitive to template sequence identity cutoff. Indeed, anticipating the results below, the cumulative fraction of cases versus predicted precision curves are very similar for all considered scenarios.

**Table 2:** Average Recall and Precision for the Various Sets of Protein Dimers^a^

Template sequence identity cutoff used	Fraction of proteins for which glue molecules are predicted	Average precision of predicted ligand	Average fraction of interface adjacent native ligands	Average of best predicted binding precision (maximum value)
**Native human protein dimers**				
No template cutoff	0.78 (2101/2682)	18%	0.76	0.24 (0.59)
30% template sequence identity cutoff	0.78 (2083)	18%	0.76	0.29 (0.59)
**E3-ligases with EGFR**				
No template cutoff	0.83 (24/29)	0.18	0.69	0.25 (0.38)
**E3-ligases with HER2**				
No template cutoff	0.98 (111/113)	0.18	0.64	0.24 (0.59)
**E3-ligases with KRAS**				
No template cutoff	0.48 (148/31)	0.20	0.80	0.21 (0.59xw)
**Diverse proteins with EGFR**				
No template cutoff	0.36(13/36)	0.18	0.83	0.23 (0.35)

a Predicted binding precision threshold of 0.15.

**Figure 5:**
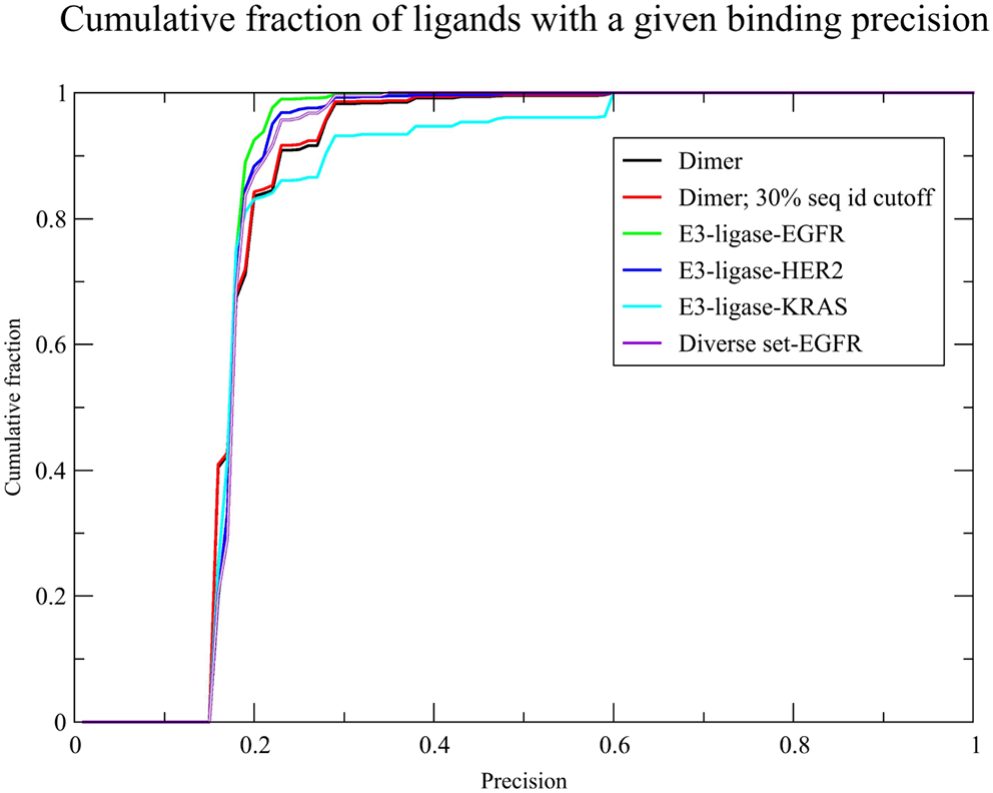
Plot of the cumulative binding precision for the six cases considered. The black line shows the set of native templates without known interfacial adjacent ligands and no template sequence identity cutoff period. The redline shows the set of native dimers with a 30% template sequence cutoff. The green line is the E3-ligase-EGFR l results. The blue line is the E3-ligase-HER2 results, the teal line is the E3-ligase- ligase results. The purple line is the diverse set-EGFR results.

We screened a library of over 1300 diverse ligands, depending on the situation. Most are not predicted to bind to the target of interest. Moreover, for the case of human dimers with no IAP ligands in their crystal structures, of the set of 37 ligands predicted with a precision >0.20, 36/37 are not known interfacial ligands. The benchmarking on the set of *E. coli* metabolites and other cases is described in.^12^ In that work and the current paper, we report the average RMSD based on the filtration criteria used to select the ligand (the *p*-value of pocket similarity and number of identical residues in the pocket alignment). In ref 12, we also show that the precision NPV, recall and RMSD of LIGMAP on 29,581 different enzymes. Thus, the strengths and weaknesses of LIGMAP have been well established and a recapitulation would be well beyond the scope of the present work.

The cutoff of the predicted binding precision that would be used to prioritize experiments is inherently context-dependent and influenced by practical considerations beyond the score itself, including the number and type of off-target interactions, drug-likeness, bioavailability, and other extrinsic constraints. As a pragmatic guideline, while higher precision is always preferable, candidates with a predicted precision of ∼20% or greater—corresponding roughly to a one-in-five chance of achieving ≤10 μM binding affinity, represent a reasonable and efficient starting point for initial experimental validation. For human dimers, one potential candidate is palmitic acid with a predicted average binding precision of 0.32. Another potential molecule of interest is pyridoxal 5′ phosphate, the active coenzyme form vitamin B6, whose predicted binding precision is 0.23. There is also a plethora of dinucleotides which also could serve as starting scaffolds for ligand design. Another interesting molecule is phycocyanobilin whose predicted binding precision is 0.21 with an average predicted binding precision. While it is known to be involved in photosynthesis, nevertheless, it might be an interesting molecule to study.

Next, we considered a subset of the 10 most frequently predicted ligands obtained without any restrictions on the relationship of the target and template proteins; all are known IAP binding ligands. Interestingly, the same top ligands are predicted even when the closest target-template sequence identity is ≤30%. Some of the top ligands are provided in Table 3. We present some of these ligands in detail as they will often be found in subsequently analyzed predictive situations involving E3-ligases as well as other classes of molecules. Thus, it is important to establish the plausibility of these results.

**Table 3:** Subset of the Frequently Selected Putative Molecular Glues for E3-Ligase with EGFR, HER2, and KRAS

Native dimers	EGFR	HER2	KRAS
**Name of ligand (Number of dimers bound)**			
HEC (Heme) (1115)^a^	OLC (oleic acid) (19)^a^	MPD2 (methyl-2–4 pentane diol) (69)^a^	MLY (*N*-methyl lysine) (89)^a^
MPD (2-methyl-2,4pentanediol) (919)^a^	LFA (eicosane) (18)^a^	CYC (phycocyanobilin) (43)	BCL (bacteriochlorophyll) (26)^a^
OLC (oleic acid) (627)^a^	BCL (bacteriochlorophyll) (18)	DMU(decyl-beta-d-maltopyranoside) (37)	MES2-(n-morpholino)-ethanesulfonic acid (18)^a^
BCR (beta-carotene) (222)	SQD (1,2-di-o-acyl-3-o-[6-deoxy-6-sulfo-alpha-d-glucopyranosyl]-sn-glycerol)(18)^a^	BCL (bacteriochlorophyll) (37)	DMUdecyl-beta-d-maltopyranoside (17)
PLM (Palmitic acid) (667)^a^	BCR(beta-carotene)(14)L2P	LMG (1,2-distearoyl-monogalactosyl-diglyceride)(15)	LDAlauryl dimethylamine-n-oxide (17)^a^
CIT (Citric acid) (526)^a^	L2P (2,3-diphytanyl-glycerol) (12)	B3K (3s)-3,7-diamino-heptanoic acid (13)	SQDsulfoquinovosyldiacylglycerol (11)
CLR (cholesterol) (452)^a^	PEK ((1s)-2-([(2-aminoethoxy)(hydroxy)phosphoryl]oxy}-1-[(stearoyloxy)methyl]ethyl (5e,8e,11e,14e)-icosa-5,8,11,14-tetraenoate) *(9)*	BLAbiliverdin ix alpha (12)	LMG1,2-distearoyl-monogalactosyl-diglyceride (11)^a^
CYC (phycocyanobilin) (431)	PLM (palmitic acid) (9)^a^	FTR Fluorotryptophane (10)	DGDdigalactosyl diacyl glycerol (10)^a^
CDL (cardiolipin) (289)^a^	TGL (tristearoylglycerol) (7)	78N ((2r)-2,3-dihydroxypropyl(7z)-pentadec-7-enoate (8)	TLATartaric acid (5)^a^
CLR (cholesterol) (15)^a^	PGV (1R-2-([([(2s)-2,3-dihydroxypropyl]oxy}(hydroxy)phosphoryl]oxy}-1-[(palmitoyloxy)methyl]ethyl (11e)-octadec-11-enoate) (6)	XCP (1S,2S)-2-aminocyclopentanecarboxylic acid (8)	BNGbeta-d-glucopyranoside (5)^a^

a Known interface adjacent binding ligand.

Among these ligands is the heme group. Hemes cause ligand-induced receptor dimerization through irreversible bis-histidine ligation. Free heme binds to the TLR4/MD2 complex and promotes TLR4 dimerization and signaling.^37^ This is a glue-like effect because the ligand stabilizes a receptor-dimer interface. Thus, there is credible evidence that heme can act *glue-like* under certain circumstances, stabilizing or inducing multimeric assemblies (e.g., in TLR4 dimers).

Another interesting case is MPD (4s-2-methyl-2,4-pentanediol). Solved protein structures provide evidence that MPD is “glue-like,” i.e., it can sit adjacent to protein–protein interfaces or even promote oligomerization.^38^ In Ca^2+^ calmodulin, an MPD molecule mediates a lattice contact by bridging helices of neighboring molecules. MPD alone (10–30% v/v) can induce heptamer formation of *Staphylococcus aureus* α-hemolysin *in vitro*; two MPD molecules bind at sites that facilitate interprotomer interactions and stabilize the pore. Thus, MPD promotes protein–protein assembly under high-concentration conditions.^39^ Furthermore, in an antibody–fluorescein Fab structure crystallized with MPD, a bound MPD molecule is trapped at the interdomain interface just below the antigen site, consistent with a small molecule occupying and effectively “gluing” an interface in its crystal structure.^40^

Evidence that oleic acid might also act as a glue is found in the interface regions of a number of solved protein structures.^11^ Another long chain fatty acid is PLM, palmitic acid. Tripalmitoylated lipopeptides glue TLR1–TLR2.^41^ The immune agonist Pam3CSK4 contains three palmitoyl chains that occupy hydrophobic pockets in both TLR2 and TLR1 which physically bridge the heterodimer. This is an archetypal “glue-like” mechanism.^41^ A C16 fatty acyl chain can bridge Frizzled receptors upon Wnt binding. The structures of Frizzled cysteine-rich domains (CRDs) bound to palmitoleic acid show that the acyl chain spans two CRD monomers, implying Wnt’s lipid *glues* a Frizzled dimer.^42^ Another study concluded that palmitic acid directly binds MD2, promotes MD2/TLR4 complex formation, and possibly triggers downstream signaling.^43^ Finally, there is a related precedent for lipid chains gluing TLR4 dimers. The TLR4–MD2–LPS cocrystal structure shows lipid A’s acyl chains filling the MD2 pocket and bridging two TLR4–MD2 complexes into the signaling-competent “M-shaped” dimer. Once again, a lipid-mediated “glue” is employed.^44^ Thus, lipids can play an important role in stabilizing protein–protein interactions

In the case of citric acid and its close derivatives, citrate is a classic allosteric activator of acetyl-CoA carboxylase (ACC1/ACC2) that induces dimerization/polymerization into active filaments.^45^ Furthermore, for β-carotene, there is direct structural evidence that it behaves as a molecular glue within native photosynthetic protein complexes, where it helps secure contacts between membrane protein subunits.^46^ β-carotene (and other carotenoids) is also a structural cofactor embedded in the membrane that stabilizes protein–protein contacts; that is, it acts very “glue-like,” in a biophysical sense.^47^

Figure 6 shows high-confidence representative examples of binding poses of small molecule ligands/that are predicted to bind in the protein interface adjacent region. Figure 6A considers the human striatin 3-coil–coil domain, PDB ID 4n6j,^48^ that is predicted to bind to tristeroglycerol (TGL). What is particularly interesting is that tristeroglycerol is a long three branched star-like molecule that literally coils around the coil–coil structure of 6n6j much like a snake constricts its prey. This suggests a possible new type of molecular glue constrictor. Figure 6B shows the binding of ERK1 (whose PDB code is 6yll^49^), that is predicted to bind to staurosporine (STU). Located in an IAP, it putatively acts to help facilitate the intermolecular interaction. Figure 6C shows the predicted binding of cardiolipin to solute-character protein 8w6h.^50^ Cardiolipin is a long-chain molecule that contains four branches, and contrary to what one might imagine that it might be fully stretched out, in practice, it folds back upon itself to fill the crevice between the interface of two proteins. More generally, there is strong structural and functional evidence that cardiolipin (CDL) molecules occupy interfaces and stabilize assemblies, notably respiratory chain supercomplexes and ATP synthase dimer. Thus, they can act as bonafide lipid glues in the inner mitochondrial membrane.^51^ Cardiolipin also binds in an interface adjacent pocket in 8ps0, an endosomal sodium/proton exchanger, whose dimeric state requires the presence of cardiolipin.^52^ Figure 6D shows the fifth bromodomain of poly bromodomain containing protein 1, 6g0j,^30^ which is predicted to bind to R78, a benzamide. For all predictions, the conformations are sterically and chemically reasonable, with ligands complementing local shape and polarity to buttress protein–protein contacts in the interface adjacent region.

**Figure 6:**
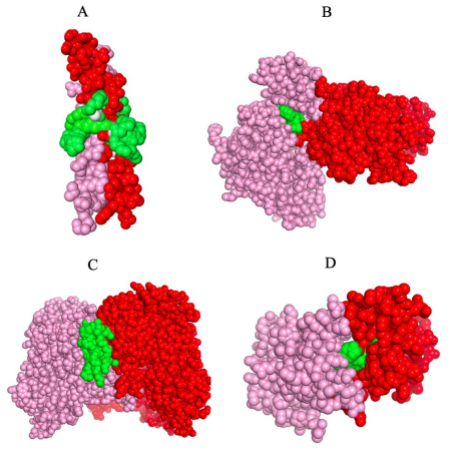
Binding poses of the ensemble of ligand conformations predicted to bind to the indicated protein dimer. Chain A is in pink, chain B in red, and the binding ligand is in green. A. Human striatin-3 coiled coil domain, 4n6j^48^ binding to TGL (tristearoylglycerol). B. ERK3, 6yll,^49^ binding to STU (staurosporine). C. Solute carrier 13, 8w6h,^50^ binding to CDL (cardiolipin). D. Fifth bromodomain of poly bromodomain containing protein 1, 6g0j,^30^ binding to R78 (4-([(7R)-8-cyclopentyl-7-ethyl-5-methyl-6-oxo-5,6,7,8-tetrahydropteridin-2-yl]­amino}-3-methoxy-N-(1-methylpiperidin-4-yl)­benzamide.

### Putative Glues between E3-Ligases EGFR, HER2, and KRAS

In this section, we restrict ourselves to the case where AF2Complex^53^ generates reasonably confident structure predictions of EGFR, HER2 or KRAS binding to an E3-ligase (IS-score >0.30). As a consequence GlueFinder’s maximal coverage is limited by the ability of AF2Complex^54^ to generate reasonably confident quaternary structure predictions.

For the case of binding EGFR to ligases, AF2Complex provides 29 predicted dimers (whose combined length is <1700 residues) that contain at least five residues on each chain that are involved in the protein–protein interface. 24/29 of these complexes have at least one predicted molecular glue whose binding precision is ≥0.15 (see Supporting Information). Furthermore, the average best binding precision is 0.24, with the best predicted ligand having a binding a precision of 0.59. The cumulative fraction of ligands with a given binding precision is shown in the green curve of Figure 5. The histogram of the set of binding ligands per protein is provided in Supporting Information. There are 167 different ligand types bound, of which 115 (69%) are known interface adjacent binding ligands indicated by “**”. The corresponding full set of binding ligands and their predicted precision per protein in is found in Supporting Information with X being the name of the protein dimer. The phylogenetic tree of the set of 24 E3 ligases is shown in Figure 7.

**Figure 7:**
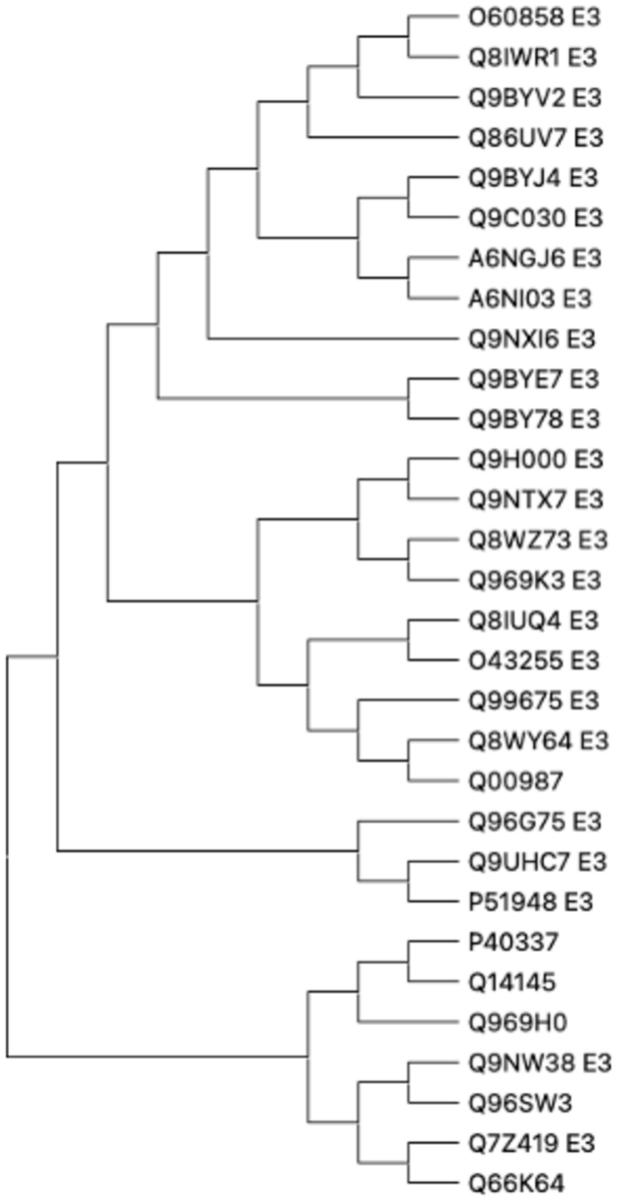
Phylogenetic tree of the 24 E3-ligases predicted to interact with EGFR assisted by a molecular glue-like metabolite are shown.

For E3-ligase-EGFR dimers, a subset of interesting putative molecular glues is presented in column 2 of Table 3. The sulfolipid SQG, (sulfoquinovosyldiacylglycerol) can play a bona fide “lipid glue” role by bridging protein subunits and stabilizing/determining dimerization in photosynthetic complexes.^55^ Higher-resolution structures reveal two SQG molecules (SQ–D1 and SQ–D2) at the monomer–monomer interface. Each forms specific hydrogen bonds to residues on different subunits across the two monomers (e.g., SQ–D1 links D1 of one monomer to CP47 of the other). Removing SQ–D breaks these bridges and shifts PSII toward monomers, i.e., it destabilizes the dimer. This is exactly a glue-like function at the protein–protein interface.^55^ Decades of PSII work conclude that bound lipids mediate protein–protein and monomer–monomer contacts within these complexes.^56^ SQD is one of the lipids repeatedly found at such oligomerization interfaces. Finally, other thylakoid lipids sometimes *are* explicitly described as a “molecular glue” (e.g., phosphatidylglycerol is proposed to glue LHCII monomers), showing that structural lipids can function as glues in native membrane assemblies.^57^

In the case of HER2, as shown in columns 2 and 3 of Table 2, GlueFinder predicts putative binding ligands for 111/113 dimers (see Supporting Information) with a predicted binding precision at or above 0.15. The cumulative fraction of ligands with a given predicted binding precision is shown in the blue curve of Figure 5. Furthermore, the histogram of the set of binding ligands per protein is provided in Supporting Information, and the corresponding full set of binding ligands and their predicted precision per protein is provided in Supporting Information with “XX” being the name of the protein dimer. A total of 237 different types of putative molecular glues is predicted with ∼64% (151/237) being known interface adjacent binding ligands. The phylogenetic tree of the 113 E3 ligases having predicted glues for HER2 along with their relationship to canonical molecular glues is shown in Figure S3.

The most frequent novel putative glue is phycocyanobilin that binds to 43/111 E3-ligase-HER2 complexes. In addition, MPD (2-methyl-2-4-pentane diol), a known interface adjacent binding ligand, is among the most frequent putative glues. MPD induces assembly of α-hemolysin into a heptamer.^39^ A study that crystallized*Staphylococcus aureus* α-hemolysin from monomeric proteins found that MPD alone (no membranes/detergent) drove formation of the SDS-resistant heptameric pore. There were two localized MPD molecules per protomer near Trp179; SDS-PAGE analysis showed efficient oligomerization. MPD facilitates and stabilizes interprotomer contacts by mimicking lipid headgroup interactions and providing a membrane-like environment. Furthermore, MPD directly participates in a protein–protein interface associated with cholera toxin B assembly.^58^ In a designed CTB pentamer system, the crystal structure revealed that MPD sites are in the interface between pentamers.^58^ Another novel ligand is LMG that can act as an IAP “glue” that stabilizes contacts between membrane–protein subunits in photosynthetic complexes. Multiple higher-resolution structures and biophysical studies show LMG molecules wedged between subunit interfaces or mediating hydrogen–bond networks that hold complexes together.^47^ Furthermore, in at least one system, BLA is “glue-like”.^59^ A cryo-EM structure of the human mitochondrial transporter ABCB10 shows BLA sitting in a pocket of one protomer and bridging the interface to the opposite protomer via hydrogen bonds, stabilizing a more closed, catalytically competent conformation. Thus, it acts as an interfacial cofactor that holds the dimer together.^59^ BLA binding also brings the two nucleotide-binding domains closer (from ∼30 Å to ∼13 Å), consistent with stabilization of the closed state.

In the case of E3 ligases putatively binding to KRAS, as shown in Table 2 148/321 predicted complexes have putative glues that are predicted to bind IAP ligands (see Supporting Information) with a precision ≥0.15, with a best average precision of 0.21. Again, we only consider predictions where at least two examples are found. The full list of putative glues for each of these 321 screened E3-ligases (having at least one example) is provided in Supporting Information. The highest precision ligand has a value of 0.59. The cumulative fraction of ligands with a given binding precision is shown in the teal curve in Figure 5. Of the 127 putative glues, 93 are known interface adjacent binding ligands, with representative examples shown in column 4 of Table 3. DMU is a novel putative glue with some literature evidence that it can help keep specific membrane protein complexes intact by occupying native lipid sites in proteins involved in the photosystem II complex.^60^ The gluing potential of SQD has been mentioned above. DGD does not yet have any approved medical uses, but does have anti-inflammatory activity in mouse models^61^ and anticancer activity/synergistic activity with doxorubicin in melanoma cells.^62^ The origin of this activity is unknown.

### Managing Large Prediction Sets through Structured Prioritization

We acknowledge that identifying dozens to hundreds of candidate E3 ligases per target (e.g., EGFR) is not intended to imply that all candidates should be experimentally tested. Rather, these predictions represent an initial hypothesis-generating layer. To translate this into a tractable experimental workflow, we propose a multitier prioritization framework: (A) Confidence Score Thresholding: Predictions can first be filtered by model confidence score. In retrospective benchmarking, we observe that higher-scoring predictions are enriched for known or structurally plausible interactions. By applying a stringent score cutoff (e.g., top 1–5% of predictions or above a calibrated probability threshold, e.g., a predicted binding precision of 0.2 or better), the candidate list can typically be reduced by an order of magnitude while maintaining high predicted precision. (B) Interface Complementarity and Structural Plausibility Filters: Candidates can be further ranked using structural metrics, such as predicted ternary complex stability (ΔΔG or interface energy), buried surface area, shape and electrostatic complementarity and predicted cooperativity of ternary complex formation. Filtering by structural plausibility substantially reduces unlikely interactions and enriches for geometrically compatible glue-mediated assemblies. (C) Biological Context Filters: We recommend incorporating biological constraints to prioritize ligases that are expressed in the relevant tissue or disease context, colocalized with the target protein (e.g., cytosolic vs membrane-associated), known to form active ubiquitination complexes in similar cellular settings and not ubiquitously essential (to avoid toxicity concerns). Public transcriptomic and proteomic data sets (e.g., TCGA, GTEx, DepMap) can be used to narrow predictions to ligases expressed in disease-relevant contexts (D) Druggability and Chemical Feasibility: Ligases can be prioritized based on the availability of structural information, the presence of ligandable pockets, prior evidence of small-molecule recruitment and availability of tool compounds or ligase binders. This further reduces the experimental space to ligases that are chemically actionable. (E) Cross-Model or Consensus Ranking: Where possible, predictions can be strengthened by agreement across independent structural modeling runs, alternative docking algorithms and orthogonal machine learning scoring functions since consensus predictions are expected to exhibit lower false positive rates. Using this multistep filtering approach, we anticipate reducing initial sets (e.g., 100+ candidates) to approximately 5–15 high-priority E3 ligases per target—well within the practical experimental scope.

### Molecular Glues for the Diverse-EGFR Set

In a harder test set, in the Diverse-EGFR set, we predicted the quaternary structure of EGFR in complex with a collection of another 25 proteins that are not E3-ligases, see Table 4. The cumulative precision of the predicted ligands is given by the purple curve of Figure 5. Here, we deliberately considered cases where *these proteins are not predicted* by AF2Complex to interact with EGFR in the absence of a ligand; rather, we are employing AF2Complex to predict interface adjacent pockets in structures which might occur with very low binding affinity. Table 4 provides an exceptionally diverse set of protein families predicted to associate through stabilization by intermolecular glues. These include homeobox and forkhead box transcription factors, aminotransferases, DNA and RNA polymerases, viral and cellular antigens, RAS-related GTPases, guanine-binding proteins, aquaporins, additional classes of transcription factors, membrane proteins, kallikreins, RNA-binding proteins, DNA–RNA-binding proteins, NF-κB-inhibitory RASD proteins, and zinc finger proteins. Collectively, this striking breadth of potential interactors suggests that EGFR can be modulated within a heterogeneous repertoire of protein contexts. Such diversity underscores the possibility of unanticipated modes of regulation and highlights the opportunities for novel therapeutic interventions, which will be elaborated upon below.

**Table 4:** Results for the Diverse-EGFR Set

UniProt ID	Protein name	IS-score	Average precision	Number of ligands^a^
A6NCQ9	RING finger protein 222 (RNF222)	0.080	0.13	28(0.79)
O15522	Homeobox protein Nkx-2.8 (NKX2–8)	0.056	0.13	350 (0.59)
O75593	Forkhead box protein H1 (FOXH1)	0.044	0.13	744 (0.41)
P09493	Ornithine aminotransferase, mitochondrial (OAT)	0.060	0.13	79 (0.60)
P0DPB6	DNA-directed RNA polymerases I and III subunit RPAC2 (POLR1D)	0.098	0.13	108 (0.78)
P20138	Myeloid cell surface antigen CD33 (CD33)	0.049	0.14	408 (0.50)
P46926	Ras GTPase-activating protein-binding protein 1 (G3BP1)	0.064	0.14	113(0.66)
P50150	Guanine nucleotide-binding protein subunit γ-4 (GNG4)	0.078	0.13	16 (0.81)
P55081	Aquaporin-1 (AQP1)	0.068	0.13	33(0.61
P59190	Ras-related protein Rab-15 (RAB15)	0.105	0.13	84(0.67)
P78545	ETS-related transcription factor Elf-3 (ELF3)	0.104	0.13	46 (0.)
Q5SZB4	Uncharacterized protein C9orf50 (C9orf50)	0.047	0.13	38 (0.71)
Q8N461	F-box/LRR-repeat protein 16 (FBXL16)	0.178	0.14	486 (0.48))
Q8NDX9	Lymphocyte antigen 6 complex locus protein G5b (LY6G5B)	0.052	0.13	109 (0.63)
Q8TAZ6	CKLF-like MARVEL transmembrane domain-containing protein 2 (CMTM2)	0.091	0.13	22 (0.69)
Q8WUH6	Transmembrane protein 263 (TMEM263)	0.055	0.13	261(0.59)
Q8WUU8	Transmembrane protein 174 (TMEM174)	0.059	0.13	29(0.79)
Q92876	Kallikrein-6 precursor (KLK6)	0.083	0.13	42 (0.76)
Q96IZ5	RNA-binding protein 41 (RBM41)	0.108	0.13	12 (0.92)
Q9GZS1	DNA-directed RNA polymerase I subunit RPA49 (POLR1E)	0.062	0.13	586 (0.46)
Q9H8G2	Caspase activity and apoptosis inhibitor 1 (CAAP1)	0.070	0.13	207 (0.63)
Q9NRB3	Carbohydrate sulfotransferase 12 (CHST12)	0.051	0.13	20 (0.80)
Q9NYR9	NF-κB inhibitor-interacting Ras-like protein 2 (NKIRAS2/KBRS2)	0.171	0.13	138 (0.70)
Q9UC06	Zinc finger protein 70 (ZNF70)	0.051	0.13	18 (0.78)
Q9Y225	RING finger protein 24 (RNF24)	0.099	0.13	119 (0.71

a Fraction of predicted glues that are native, interface adjacent metabolites.

From Table 4, the average predicted precision is 0.18, with 83% of the predicted putative glues in the native IAP ligand binding library. The average of the best predicted binding precision is 0.23, with the absolute best predicted binding precision of 0.35. The total set of binding ligands per protein is provided in Supporting Information. As previously, ligands not in the native IAP ligand library include CYC (phycocyanobilin), BCL (bacteriochlorophyll), BCR (beta-carotene), GLY (glycine), UMP (5-deoxyuridine 5′-monophosphate), SQD (1,2-di-o-acyl-3-o-[6-deoxy-6-sulfo-alpha-d-glucopyranosyl]-*sn*-glycerol) and AK6 (2-oxoglutaric acid).

### Examination of EGFR Binding to Canonical E3 Ligases Having Known Molecular Glues to Other Protein Targets

For EGFR, none of the four E3 ligases which have molecular glues to other targets, (Cereblon (Q96SW2),^63^ VHL (P40337),^64^ MDM2 (Q00987),^65^ DCAF15 (Q66K64),^66^ ) are predicted by AF2Complex to form stable dimers without a molecular glue. For Cereblon, citric acid (SAH, adenosyl homocysteine) is predicted to be a molecular glue with EGFR with a precision of 0.21 (0.19). For VHL, NAG, 2-acetamido-2-deoxy-beta-d-glucopyranose (BCR, beta-carotene), is predicted to be a molecular glue with EGFR with a precision of 0.29 (0.17). For MDM2, Y01, cholesterol hemisuccinate (OLC, 2R-2,3-dihydroxypropyl (9Z)-octadec-9-enoate) is predicted to act as a molecular glue with EGFR with a precision of 0.18 (0.17). For DCAF5, a top putative molecular glue is CIT (BTB, 2-[bis­(2-hydroxy-ethyl)-amino]-2-hydroxymethylpropane-1,3-diol) with a predicted binding precision of 0.19 (0.14).

## Discussion

The development of molecular glue degraders opens a transformative avenue in targeted protein degradation, enabling modulation of previously “undruggable” proteins by promoting proximity-induced ubiquitination and subsequent proteasomal degradation. Despite their therapeutic promise, molecular glues have largely been discovered serendipitously, with rational design approaches still in their infancy. A major bottleneck is the field’s overreliance on a narrow set of E3 ligases, primarily Cereblon (CRBN) and von Hippel–Lindau (VHL), which limits the diversity of targetable substrates and creates resistance risks through ligase saturation or mutation. Expanding the set of proteins beyond these traditional ligases is essential for fully realizing the potential of molecular glues as a generalizable therapeutic modality.

To achieve this, there is a critical need for a rational, unbiased platform that can systematically explore the entire E3 ligase landscape and identify potential molecular glue opportunities across a broad range of protein targets. One step in this direction is the global structural and statistical analysis of protein–protein interfaces and their adjacent ligand-binding events across the PDB. By identifying ligands that naturally localize near protein–protein interfaces—particularly those that bridge or stabilize two proteins-one might infer structural and chemical principles that favor ternary complex formation. These protein interface adjacent ligand-bound pockets often represent possible opportunities for molecular glue design, even if they were not originally identified in the context of degradation.

The ligand-centric, interface-aware strategy of GlueFinder provides a scalable means to prioritize small molecules and protein pairs for experimental validation, offering a contrast to trial-and-error screening approaches. Importantly, this approach is agnostic to the identity of the E3 ligase, enabling exploration of the full human E3 ligase repertoire—over 600 ligases—rather than restricting design to a few well-characterized examples. This not only diversifies potential degradation mechanisms but also opens avenues for tissue-specific or disease-selective targeting by leveraging E3 ligase expression patterns.

Recent advances in structural biology, particularly AlphaFold 2,^67^ AlphaFold-Multimer,^68^ AF2Complex,^54^ AlphaFold 3 ^69^ and AF3Complex^70^ now enable high-confidence predictions of quaternary protein structures at scale, including E3-ligase complexes for which no experimental structure exists. Moreover, even when a given set of proteins are not predicted to form inherently *stable* dimers, these algorithms sometimes generate reasonable structures to which a ligand could bind to its interface adjacent pocket, thereby stabilizing the complex. This after all is the goal of molecular glues. Integrating predicted POI–E3 complexes with statistical maps of interface-adjacent ligandability allows researchers to computationally screen for “druggable” interfacial pockets across theoretical ternary assemblies. This empowers a rational framework for glue design even when direct structural data is lacking, significantly expanding the scope of targetable interactions.

Our putative EGFR diverse protein interactors span transcriptional regulators (ETS-family factors and zinc-finger proteins), protein quality control proteins (RING-type E3 ligases), vesicle trafficking proteins (the RAS-related GTPase RAB5), and even DNA-associated enzymes (including polymerases). This diversity suggests that interface-adjacent small molecules (“molecular glues”) could be leveraged not only to stabilize cryptic EGFR complexes but also to reroute the information flow across levels of regulation—transcription, membrane trafficking, and proteostasis. For example, glues that couple ETS factors or other coactivators to EGFR-proximal chromatin machinery could upregulate EGFR expression under physiological, nononcogenic contexts (e.g., tissue repair), whereas glues that recruit RING E3-ligases to EGFR would favor ligase-directed ubiquitylation and turnover. Likewise, glues that bias EGFR’s endocytic itinerary via RAB5-proximal effectors could retune receptor surface dwell time and signaling duration. Conceptually, this extends synthetic-biology paradigms—where small molecules have been used to retask protein assemblies—to reengineer native mammalian complexes *in situ* for programmable gain- or loss-of-function phenotypical function.

We further note that the absence of supporting literature does not necessarily invalidate the predicted set of molecules as possible molecular glues. Rather, it may reflect their novelty and offers the potential to uncover previously unrecognized molecular glues. At the same time, one must acknowledge the possibility of overprediction, wherein certain compounds bind so weakly that they fail to exert a meaningful structural or functional effect. In such cases, the ligand may not induce dimerization. Thus, while some predictions may represent bona fide novel glues, others may prove nonfunctional, underscoring the need for careful experimental validation.

Broadly speaking, two principal classes of molecular glues can be considered. The first comprises the so-called bridging compounds, here referred to as interface-adjacent pocket (IAP) ligands, which occupy the interfacial region between two interacting proteins and directly stabilize their association. The second class consists of allosteric glues, which act at a distance by inducing conformational rearrangements that expose previously buried hydrophobic surfaces, thereby promoting new protein–protein interactions. While the latter mechanism cannot be excluded, the current class of algorithms is specifically designed to identify the former type—that is, ligands residing in or near the protein–protein interface. Interestingly, even in systems such as CRBN, where one domain sometimes undergoes partial opening upon ligand binding, the ligand-binding domain itself remains largely structurally conserved, functioning effectively as a rigid body whose ligand pose is structurally conserved consistent with the assumptions underlying GlueFinder. Although the present framework does not explicitly predict domain cracking or other large-scale rearrangements—tasks more suited to structure prediction algorithms of the AlphaFold class—it remains applicable in cases where the rearranged domain still engages its partner through an interfacial or interface-adjacent ligand. Thus, while this approach does not yet provide a complete solution to the full spectrum of molecular glue mechanisms, it represents a step forward. Ongoing efforts aim to extend the method to predict allosteric conformational transitions, thereby addressing scenarios where ligand binding indirectly promotes or stabilizes protein–protein association.

Our work, by integrating global structural analysis with AI-powered structure prediction, provides a rational, predictive approach toward molecular glue prioritization-one that does not depend on chance or historical bias. This paradigm shift holds the potential to unlock new degrader targets, harness underutilized E3 ligases, and create a scalable, mechanism-informed path for therapeutic development. It is through this convergence of computational insight and structural understanding followed ultimately by experimental validation that the full power of molecular glue degraders could be harnessed across the proteome.

## Materials and Methods

A schematic overview of the GlueFinder methodology is shown in Figure 8. In practice, GlueFinder was applied under two scenarios: (1). For cases with preexisting experimental dimeric structures in the PDB, interface-adjacent pockets are identified and characterized using the Cavitator pocket alignment algorithm.^25^ (2). For a pair of molecules whose quaternary structure is unknown, the structure of their complex is first predicted using AF2Complex (AF2C).^54^ Even when a confident protein–protein interaction cannot be predicted, we empirically observed that AF2C frequently generates reasonable interface-adjacent pocket poses suitable for small molecule screening. This reflects the fact that the library of protein pockets is finite (about 500 distinct clusters) and is a feature of protein structures alone.^13,25^ Thus, if a pair of proteins weakly interacts and provides a given IAP, then a ligand which binds to this IAP could stabilize this complex. The question then is not whether the IAP consistent with this pose is physical, but whether the free energy of this pose is sufficiently favorable that on binding to the molecular glue, it will be sufficiently populated.

**Figure 8:**
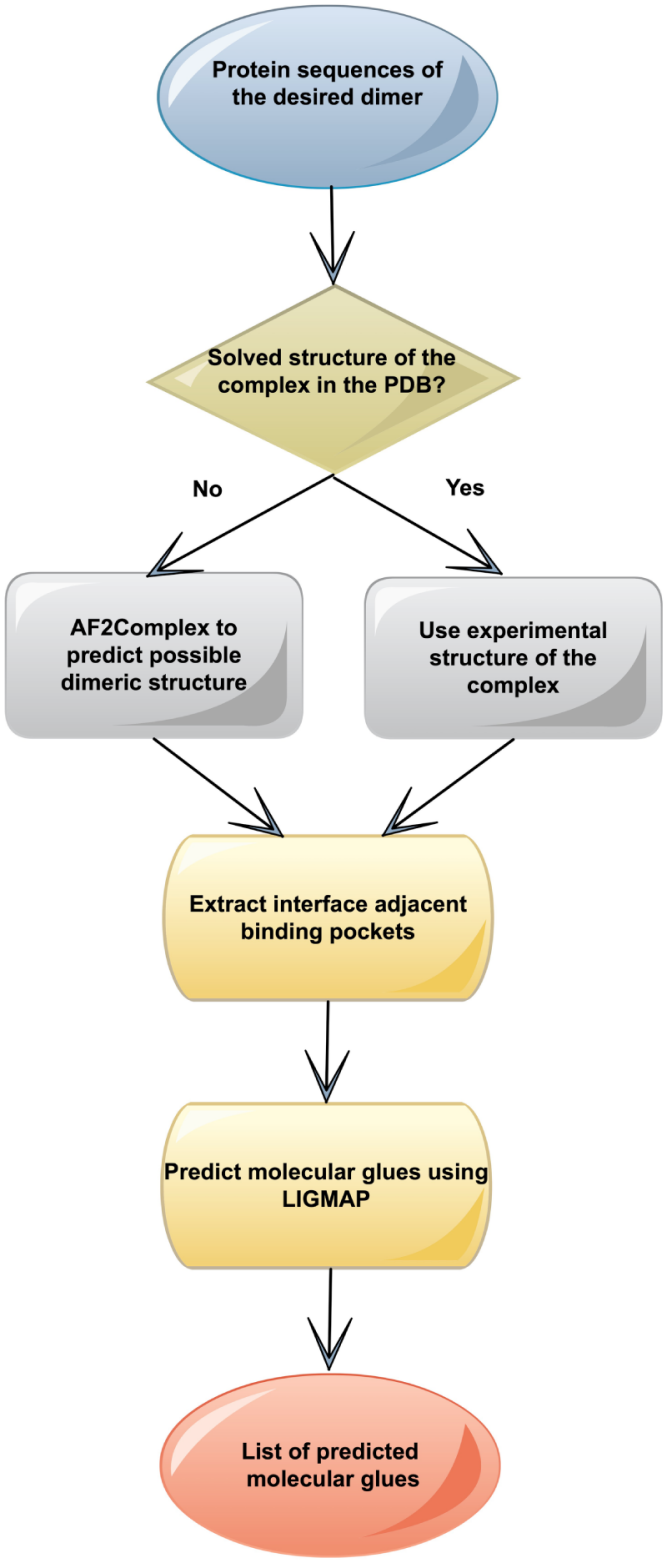
Overview of the GlueFinder molecular glue prediction algorithm.

This scenario is particularly relevant for the design of molecular glues, where the interacting partners are unlikely to associate without a third molecule that stabilizes the complex and shifts the binding free energy toward the bound state. For both scenarios, IAPs are then identified using Cavitator. Subsequently, the APoc protein pocket structure alignment algorithm^25^ is applied to screen a library of small molecule pockets found in the entire Protein Data Bank (PDB). Candidate complexes are then ranked and subjected to virtual screening with the LIGMAP algorithm^12^ which finds stereochemically similar pockets that bind the set of ligands in the given ligand library. LIGMAP works by first calculating the p-value of the pocket similarity between the target structure and the template pocket that binds a given ligand. It then counts the number if identical residues in the pocket alignment. The template ligand is then transferred to the target pocket based on the alignment provide by APoc.

Based on prior retrospective benchmarking against known structures as well as experimental ligand protein complexes,^12,71^ the precision that a given ligand is likely to bind with at worst a 10 μM binding affinity is then provided.^12^ There the precision, recall and negative predictive value (NPV) were shown for the case of 10 different metabolites in *E. coli*. At a predicted binding precision of 0.3, the NPV is 0.4. For cases with a predicted precision of 0.6, the NPV is 0.6. In practice, the predicted precision (obtained from benchmarking) depends on the p-value of pocket similarity and the number of identical aligned residues found in the target and template pockets (see below). But in practice if 10 or more ligands are screened at least 2 hits should be found.

### Native Interface Adjacent Binding Ligands

The entire Protein Data Bank (PDB) from September 2024 was scanned to identify dimeric complexes, irrespective of whether they were homodimers or heterodimers. For this analysis, only dimers were considered, yielding a total of 63,091 cases (see SI LIST.fullpdb_chains_mult.dimers). To ensure that the interactions between chains represented genuine dimeric contacts, we required that each chain contribute at least five interacting residues to its partner chain. This criterion was chosen to provide a minimal likelihood that the observed interfaces correspond to physiologically meaningful interactions. From this curated set of dimers, we extracted all bound ligands and then restricted the analysis to those ligands that contacted at least five residues on each chain of the dimer. Application of these filters resulted in a set of 1,665 ligands, which are provided in the Supporting Information; the dimeric proteins that contributed to this list are found in Supporting Information.

### Library of Solved Human Protein Structures

All dimeric, human protein complexes in the Protein Data Bank (PDB) were extracted, yielding 15,680 structures. Of these, 238 are protein complexes with known interface adjacent binding ligands (see Supporting Information). This “native benchmark” set will be used to establish the precision and recall of GlueFinder as well as its ability to correctly position ligands in the interface adjacent pocket. To reduce redundancy and generate representative data sets, the full set of human PDB dimers were clustered at 70% sequence identity, yielding 2,695 complexes; see Supporting Information. Of these, 2,563 have ligand binding predictions at a template sequence identity cutoff of 30%. For most proteins, there are no known molecular glues. These were used to test the ability of GlueFinder to identify possible small molecule glues in a comprehensive set of human protein dimers.

### Small Molecule Screening Library

All small ligands in the September 2024 Protein Data Bank (found in individual chains) were systematically extracted, and a representative library was constructed from pockets containing at least 10 amino acid residues and a minimum volume of 100 Å^3^; thereby ultra shallow glues and bridging glues with minimal protein contacts will be missed. However, such glues might have weak binding affinity with possibly small physiological consequences.

This procedure yielded 1,314 distinct ligands, which are provided in Supporting Information. The corresponding ligand–pocket PDB associations are in Supporting Information which contains 94,074 structures. The distribution of the distinct ligands across pockets is highly variable, ranging from 99 instances for YCM (S-(2-amino-2-oxyethyl)-l-cysteine) to a single occurrence for ligands such as 289 (d-glycero-alpha-d-manno-heptopyranose).^72^

### Prediction of Molecular Glues between E3-Ligases and EGFR, HER2, and KRAS

A list of 390 human E3-ligases from NHLBI (see Supporting Information) was used to predict high confidence predictions of the complexes with EGFR, HER2 and KRAS using AF2Complex.^54^ None of the considered complexes had solved PDB structures.

## Software and Data Availability

The necessary input files and output files are all provided in Supporting Information at https://github.com/hzhou3ga/gluefinder. The source code is free for academic use and evaluation purposes.

## Supplementary Material





## Notes

Figure S1: the structure of 8tnp interacting with DNA damage protein 1 and pomalidomide—an IAP binding ligand (upper); 8tnp and the superposition onto the monomeric 8rq8A (lower); Figure S2: the structural alignment of CRBN in 8rq8A and 6hf0B, where the pomalidomide binding domain opens and detaches from the remainder of the protein to interact with IKAROS; Figure S3: the phylogenetic tree of the 113 E3 ligases having predicted glues for HER2, along with their relationship to canonical molecular glues (PDF)
(ZIP)

## Abbreviations

MGD: molecular glue degraders
PROTAC: proteolysis-targeting chimeras

## References

[1] Yu S., Zhou L., Yang J., Zhang J., Lu W. (2025). Exploring
nondegrading molecular glues for protein-protein interactions. Trends Biochem. Sci..

[2] Zhou Q. Q., Xiao H. T., Yang F., Wang Y. D., Li P., Zheng Z. G. (2023). Advancing targeted
protein degradation for metabolic
diseases therapy. Pharmacol. Res..

[3] Yang Y., Yao Q., Song D., Tang K., Tang Z., Hu M., Luo Y., Xie Y. (2025). Discovery of degrader for FLT3, GSPT1 and IKZF1/3 proteins
merging PROTAC and molecular glue targeting FLT3-ITD mutant acute
myeloid leukemia. Eur. J. Med. Chem..

[4] Dewey J. A., Delalande C., Azizi S. A., Lu V., Antonopoulos D., Babnigg G. (2023). Molecular Glue Discovery: Current and Future Approaches. J. Med. Chem..

[5] Holdgate G. A., Bardelle C., Berry S. K., Lanne A., Cuomo M. E. (2024). Screening
for molecular glues - Challenges and opportunities. SLAS Discovery.

[6] Rui H., Ashton K. S., Min J., Wang C., Potts P. R. (2023). Protein-protein
interfaces in molecular glue-induced ternary complexes: Classification,
characterization, and prediction. RSC Chem.
Biol..

[7] Mayor-Ruiz C., Bauer S., Brand M., Kozicka Z., Siklos M., Imrichova H., Kaltheuner I. H., Hahn E., Seiler K., Koren A. (2020). Rational
discovery of molecular glue degraders via scalable chemical profiling. Nat. Chem. Biol..

[8] Weiss D. R., Bortolato A., Sun Y., Cai X., Lai C., Guo S., Shi L., Shanmugasundaram V. (2023). On Ternary Complex Stability in Protein
Degradation: In Silico Molecular Glue Binding Affinity Calculations. J. Chem. Inf. Model..

[9] Toriki E. S., Papatzimas J. W., Nishikawa K., Dovala D., Frank A. O., Hesse M. J., Dankova D., Song J. G., Bruce-Smythe M., Struble H. (2023). Correction
to “Rational Chemical Design of Molecular Glue Degraders”. ACS Cent. Sci..

[10] Petzold G., Gainza P., Annunziato S., Lamberto I., Trenh P., McAllister L. A., De Marco B., Schwander L., Bunker R. D., Zlotosch M. (2025). Mining the CRBN target
space redefines rules for molecular glue-induced neosubstrate recognition. Science.

[11] Skolnick J., Zhou H. (2022). Implications of the
Essential Role of Small Molecule Ligand Binding
Pockets in Protein-Protein Interactions. J.
Phys. Chem. B.

[12] Skolnick J., Srinivasan B., Skolnick S., Zhou H. (2025). Entabolons: How metabolites
modify the biochemical function of proteins and cause the correlated
behavior of proteins in pathways. J. Chem. Inf.
Model..

[13] Gao M., Skolnick J. (2013). A comprehensive survey of small-molecule binding pockets
in proteins. PLoS Comput. Biol..

[14] Kang C. B., Xu W. J. (2025). Leveraging Structural
and Computational Biology for Molecular Glue
Discovery. J. Med. Chem..

[15] Choi T. S., Tezcan F. A. (2022). Overcoming universal
restrictions on metal selectivity
by protein design. Nature.

[16] Gao M., Zhou H., Skolnick J. (2015). Insights into
Disease-Associated
Mutations in the Human Proteome through Protein Structural Analysis. Structure.

[17] Timasheff S. N. (1998). Control
of protein stability and reactions by weakly interacting cosolvents:
The simplicity of the complicated. Adv. Protein
Chem..

[18] Bukhdruker S., Varaksa T., Orekhov P., Grabovec I., Marin E., Kapranov I., Kovalev K., Astashkin R., Kaluzhskiy L., Ivanov A. (2023). Structural
insights into the effects of glycerol on ligand binding to cytochrome
P450. Acta Crystallogr. D Struct Biol..

[19] Dietary Reference Intakes for Thiamin, Riboflavin, Niacin, Vitamin B(6), Folate, Vitamin B(12)Pantothenic Acid, Biotin, and Choline; The National Academies Collection: Reports funded by National Institutes of Health, National Academies Press: US, 1998. 23193625

[20] Laganowsky A., Reading E., Allison T. M., Ulmschneider M. B., Degiacomi M. T., Baldwin A. J., Robinson C. V. (2014). Membrane
proteins bind lipids selectively to modulate their structure and function. Nature.

[21] Vallee B. L., Auld D. S. (1990). Zinc coordination,
function, and structure of zinc enzymes and other proteins. Biochemistry.

[22] Silva, J. J. R. ; Williams, R. J. P. The biological chemistry of the elements: The inorganic chemistry of life; Oxford University Press, 2001.

[23] Osman D., Robinson N. J. (2023). Protein metalation
in a nutshell. FEBS Lett..

[24] Daylight Theory Manual; Daylight Chemical Information Systems, Inc.; Daylight Chemical Information Systems, Inc: Aliso Viejo, CA, 2007. http://www.daylight.com.

[25] Gao M., Skolnick J. (2013). APoc: large-scale identification
of similar protein
pockets. Bioinformatics.

[26] Furihata H., Yamanaka S., Honda T., Miyauchi Y., Asano A., Shibata N., Tanokura M., Sawasaki T., Miyakawa T. (2020). Structural
bases of IMiD selectivity that emerges by 5-hydroxythalidomide. Nat. Commun..

[27] Kroupova A., Spiteri V. A., Rutter Z. J., Furihata H., Darren D., Ramachandran S., Chakraborti S., Haubrich K., Pethe J., Gonzales D. (2024). Design
of a Cereblon construct for crystallographic
and biophysical studies of protein degraders. Nat. Commun..

[28] Fischer E. S., Bohm K., Lydeard J. R., Yang H., Stadler M. B., Cavadini S., Nagel J., Serluca F., Acker V., Lingaraju G. M. (2014). Structure of the DDB1-CRBN E3 ubiquitin ligase
in complex with thalidomide. Nature.

[29] Bouguenina H., Scarpino A., O’Hanlon J. A., Warne J., Wang H. Z., Wah Hak L. C., Sadok A., McAndrew P. C., Stubbs M., Pierrat O. A. (2023). A Degron Blocking Strategy Towards Improved
CRL4­(CRBN) Recruiting PROTAC Selectivity. ChemBiochem.

[30] Burley S. K., Piehl D. W., Vallat B., Zardecki C. (2024). RCSB Protein Data Bank:
Supporting research and education worldwide through explorations of
experimentally determined and computationally predicted atomic level
3D biostructures. IUCrJ..

[31] Petzold G., Fischer E. S., Thoma N. H. (2016). Structural
basis of lenalidomide-induced
CK1alpha degradation by the CRL4­(CRBN) ubiquitin ligase. Nature.

[32] Matyskiela M. E., Lu G., Ito T., Pagarigan B., Lu C. C., Miller K., Fang W., Wang N. Y., Nguyen D., Houston J. (2016). A novel
cereblon modulator recruits GSPT1 to the CRL4­(CRBN) ubiquitin
ligase. Nature.

[33] Sievers Q. L., Petzold G., Bunker R. D., Renneville A., Slabicki M., Liddicoat B. J., Abdulrahman W., Mikkelsen T., Ebert B. L., Thoma N. H. (2018). Defining
the human
C2H2 zinc finger degrome targeted by thalidomide analogs through CRBN. Science.

[34] Ma X., Leon B., Ornelas E., Dovala D., Tandeske L., Luu C., Pardee G., Widger S., Solomon J. M., Beckwith R. E. J. (2023). Structural and biophysical comparisons of the pomalidomide-
and CC-220-induced interactions of SALL4 with cereblon. Sci. Rep..

[35] Mercer J. A. M., De Carlo S. J., Roy Burman S. S., Sreekanth V., Nelson A. T., Hunkeler M., Chen P. J., Donovan K. A., Kokkonda P., Tiwari P. K. (2024). Continuous
evolution
of compact protein degradation tags regulated by selective molecular
glues. Science.

[36] a Srinivasan, B. ; Skolnick, J. ; Zhou, H. Molecules with potent DHFR binding affinity and antibacterial activity; US 9,920,058 B2, 2014.

[37] Belcher J. D., Zhang P., Nguyen J., Kiser Z. M., Nath K. A., Hu J., Trent J. O., Vercellotti G. M. (2020). Identification
of a Heme Activation Site on the MD-2/TLR4
Complex. Front. Immunol..

[38] Lin J., van den Bedem H., Brunger A. T., Wilson M. A. (2016). Atomic resolution
experimental phase information reveals extensive disorder and bound
2-methyl-2,4-pentanediol in Ca 2+ -calmodulin. Acta Crystallogr. D Struct Biol..

[39] Tanaka Y., Hirano N., Kaneko J., Kamio Y., Yao M., Tanaka I. (2011). 2-Methyl-2,4-pentanediol induces spontaneous assembly
of staphylococcal alpha-hemolysin into heptameric pore structure. Protein Sci..

[40] Herron J. N., He X. M., Mason M. L., Voss Jr E. W., Edmundson A. B. (1989). Three-dimensional
structure of a fluorescein-Fab complex crystallized in 2-methyl-2,4-pentanediol. Proteins.

[41] Jin M. S., Kim S. E., Heo J. Y., Lee M. E., Kim H. M., Paik S. G., Lee H., Lee J. O. (2007). Crystal structure
of the TLR1-TLR2 heterodimer induced by binding of a tri-acylated
lipopeptide. Cell.

[42] Nile A. H., Mukund S., Stanger K., Wang W., Hannoush R. N. (2017). Unsaturated
fatty acyl recognition by Frizzled receptors mediates dimerization
upon Wnt ligand binding. Proc. Natl. Acad. Sci.
U. S. A..

[43] Wang Y., Qian Y., Fang Q., Zhong P., Li W., Wang L., Fu W., Zhang Y., Xu Z., Li X. (2018). Author
Correction: Saturated palmitic acid induces myocardial inflammatory
injuries through direct binding to TLR4 accessory protein MD2. Nat. Commun..

[44] Park B. S., Song D. H., Kim H. M., Choi B. S., Lee H., Lee J. O. (2009). The structural basis
of lipopolysaccharide recognition
by the TLR4-MD-2 complex. Nature.

[45] Zhou F., Zhang Y., Zhu Y., Zhou Q., Shi Y., Hu Q. (2024). Filament structures unveil the dynamic organization of human acetyl-CoA
carboxylase. Sci. Adv..

[46] Pan X., Cao D., Xie F., Xu F., Su X., Mi H., Zhang X., Li M. (2020). Structural
basis for electron transport
mechanism of complex I-like photosynthetic NAD­(P)H dehydrogenase. Nat. Commun..

[47] Schuller J. M., Birrell J. A., Tanaka H., Konuma T., Wulfhorst H., Cox N., Schuller S. K., Thiemann J., Lubitz W., Setif P. (2019). Structural adaptations
of photosynthetic complex I enable ferredoxin-dependent
electron transfer. Science.

[48] Chen C., Shi Z., Zhang W., Chen M., He F., Zhang Z., Wang Y., Feng M., Wang W., Zhao Y. (2014). Striatins
contain a noncanonical coiled coil that binds protein phosphatase
2A A subunit to form a 2: 2 heterotetrameric core of striatin-interacting
phosphatase and kinase (STRIPAK) complex. J.
Biol. Chem..

[49] Gradler U., Busch M., Leuthner B., Raba M., Burgdorf L., Lehmann M., Linde N., Esdar C. (2021). Corrigendum to “Biochemical, cellular and structural characterization
of novel and selective ERK3 inhibitors [Bioorg. Med. Chem. Lett. 30
(2020) 127551]”. Bioorg. Med. Chem. Lett..

[50] Chi X., Chen Y., Li Y., Dai L., Zhang Y., Shen Y., Chen Y., Shi T., Yang H., Wang Z. (2024). Cryo-EM structures of
the human NaS1 and NaDC1 transporters
revealed the elevator transport and allosteric regulation mechanism. Sci. Adv..

[51] Bazán S., Mileykovskaya E., Mallampalli V. K. P.
S., Heacock P., Sparagna G. C., Dowhan W. (2013). Cardiolipin-dependent Reconstitution
of Respiratory Supercomplexes from Purified Complexes III and IV. J. Biol. Chem..

[52] Kokane S., Gulati A., Meier P. F., Matsuoka R., Pipatpolkai T., Albano G., Ho T. M., Delemotte L., Fuster D., Drew D. (2025). PIP­(2)-mediated oligomerization
of
the endosomal sodium/proton exchanger NHE9. Nat. Commun..

[53] Feldman J., Skolnick J. (2025). AF3Complex yields improved structural predictions of
protein complexes. Bioinformatics.

[54] Gao M., Nakajima an D., Parks J. M., Skolnick J. (2022). AF2Complex predicts
direct physical interactions in multimeric proteins with deep learning. Nat. Commun..

[55] Nakajima Y., Umena Y., Nagao R., Endo K., Kobayashi K., Akita F., Suga M., Wada H., Noguchi T., Shen J. R. (2018). Thylakoid membrane lipid sulfoquinovosyl-diacylglycerol
(SQDG) is required for full functioning of photosystem II in Thermosynechococcus
elongatus. J. Biol. Chem..

[56] Loll B., Kern J., Saenger W., Zouni A., Biesiadka J. (2007). Lipids in
photosystem II: Interactions with protein and cofactors. Biochim. Biophys. Acta.

[57] Flachmann R., Kuhlbrandt W. (1996). Crystallization
and identification of an assembly defect
of recombinant antenna complexes produced in transgenic tobacco plants. Proc. Natl. Acad. Sci. U. S. A..

[58] Ross J. F., Wildsmith G. C., Johnson M., Hurdiss D. L., Hollingsworth K., Thompson R. F., Mosayebi M., Trinh C. H., Paci E., Pearson A. R. (2019). Directed Assembly of Homopentameric Cholera
Toxin B-Subunit Proteins into Higher-Order Structures Using Coiled-Coil
Appendages. J. Am. Chem. Soc..

[59] Cao S., Yang Y., He L., Hang Y., Yan X., Shi H., Wu J., Ouyang Z. (2023). Cryo-EM structures of mitochondrial
ABC transporter ABCB10 in apo and biliverdin-bound form. Nat. Commun..

[60] Sakurai I., Mizusawa N., Wada H., Sato N. (2007). Digalactosyldiacylglycerol
is required for stabilization of the oxygen-evolving complex in photosystem
II. Plant Physiol..

[61] Bruno A., Rossi C., Marcolongo G., Di Lena A., Venzo A., Berrie C. P., Corda D. (2005). Selective
in vivo anti-inflammatory
action of the galactolipid monogalactosyldiacylglycerol. Eur. J. Pharmacol..

[62] Grabowska K., Galanty A., Koczurkiewicz-Adamczyk P., Wrobel-Biedrawa D., Zmudzki P., Zaluski D., Wojcik-Pszczola K., Pasko P., Pekala E., Podolak I. (2021). Multidirectional anti-melanoma
effect of galactolipids (MGDG-1 and DGDG-1) from Impatiens parviflora
DC. and their synergy with doxorubicin. Toxicol.
In Vitro.

[63] Sasso J. M., Tenchov R., Wang D., Johnson L. S., Wang X., Zhou Q. A. (2023). Molecular Glues:
The Adhesive Connecting Targeted Protein
Degradation to the Clinic. Biochemistry.

[64] Tutter A., Buckley D., Golosov A. A., Ma X., Shu W., McKay D. J. J., Darsigny V., Dovala D., Beckwith R., Solomon J. (2025). A small-molecule VHL molecular glue degrader for cysteine
dioxygenase 1. Nat. Chem. Biol..

[65] Wang B., Liu J., Tandon I., Wu S., Teng P., Liao J., Tang W. (2021). Development of MDM2
degraders based on ligands derived from Ugi reactions:
Lessons and discoveries. Eur. J. Med. Chem..

[66] Nijhuis A., Sikka A., Yogev O., Herendi L., Balcells C., Ma Y., Poon E., Eckold C., Valbuena G. N., Xu Y. (2022). Indisulam
targets RNA splicing and metabolism to serve as a therapeutic
strategy for high-risk neuroblastoma. Nat. Commun..

[67] Jumper J., Evans R., Pritzel A., Green T., Figurnov M., Ronneberger O., Tunyasuvunakool K., Bates R., Zidek A., Potapenko A. (2021). Highly accurate protein structure prediction
with AlphaFold. Nature.

[68] Evans, R. ; O’Neill, M. ; Pritzel, A. ; Antropova, N. ; Senior, A. ; Green, T. ; Žídek, A. ; Bates, R. ; Blackwell, S. ; Yim, J. Protein complex prediction with AlphaFold-Multimer bioRxiv 2022 10.1101/2021.10.04.463034

[69] Roy R., Al-Hashimi H. M. (2024). AlphaFold3
takes a step toward decoding molecular behavior
and biological computation. Nat. Struct Mol.
Biol..

[70] Feldman J., Skolnick J. (2025). AF3Complex Yields Improved Structural
Predictions of
Protein Complexes. Bioinformatics.

[71] Piazza I., Kochanowski K., Cappelletti V., Fuhrer T., Noor E., Sauer U., Picotti P. (2018). A Map of Protein-Metabolite Interactions
Reveals Principles of Chemical Communication. Cell.

[72] McGregor A., Klartag M., David L., Adir N. (2008). Allophycocyanin trimer
stability and functionality are primarily due to polar enhanced hydrophobicity
of the phycocyanobilin binding pocket. J. Mol.
Biol..

