# Supplementary material for: GlueFinder: A Data-Driven Framework for the Rational Discovery of Molecular Glues
Source: J Chem Inf Model. 2026 Mar 3;66(6):3368–82. doi: 10.1021/acs.jcim.5c03232 (PMC13014457; doi:10.1021/acs.jcim.5c03232)
Supplement: Supplementary file 1 [file ci5c03232_si_001.pdf]

GlueFinder: A Data-Driven Framework for the Rational Discovery of Molecular Glues

Jeffrey Skolnick[1*], Bharath Srinivasan[2,3,4,5], and Hongyi Zhou[1]

[1]Center for the Study of Systems Biology, Georgia Institute of Technology, 950 Atlantic Dr NW, Atlanta, GA 30332, USA

[2]School of Pharmacy and Life Sciences, Robert Gordon University, Garthdee House, Garthdee Rd, Aberdeen AB10 7AQ, UK

[3]Department of Chemistry and [4]Center for the Advanced Study of Drug Action, Stony Brook University, Stony Brook NY, 11794-3400, USA

[5]Cancer Research Horizons, Cancer Research UK, 2 Redman Place. London. E20 1JQ, UK

Corresponding author:

* skolnick@gatech.edu

Supporting Information

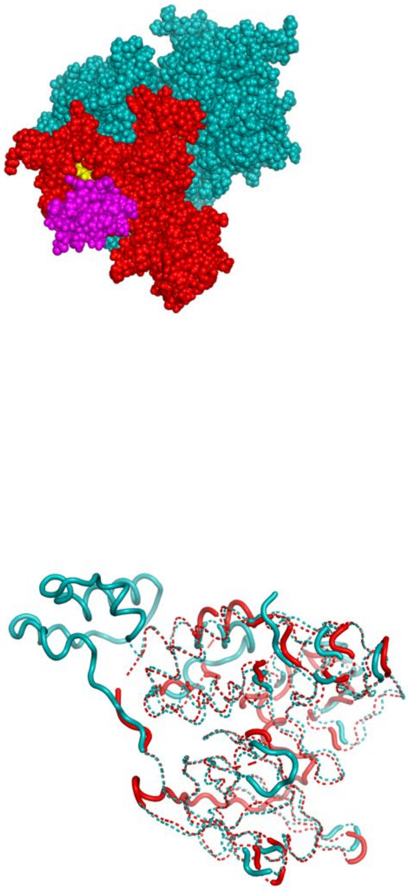

Fig. S1. The structure of 8tnp interacting with DNA damage protein 1 and pomalidomide-an IAP binding ligand (upper); 8tnp and the superposition onto the monomeric 8rq8A (lower).

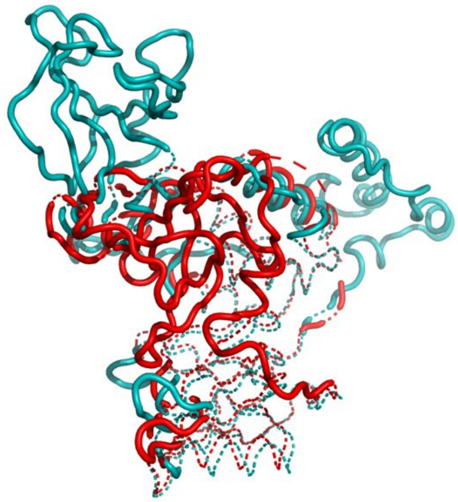

Fig. S2. The structural alignment of CRBN in 8rq8A and 6hf0B where the pomalidomide binding domain opens and detaches from the remainder of the protein to interact with IKAROS.

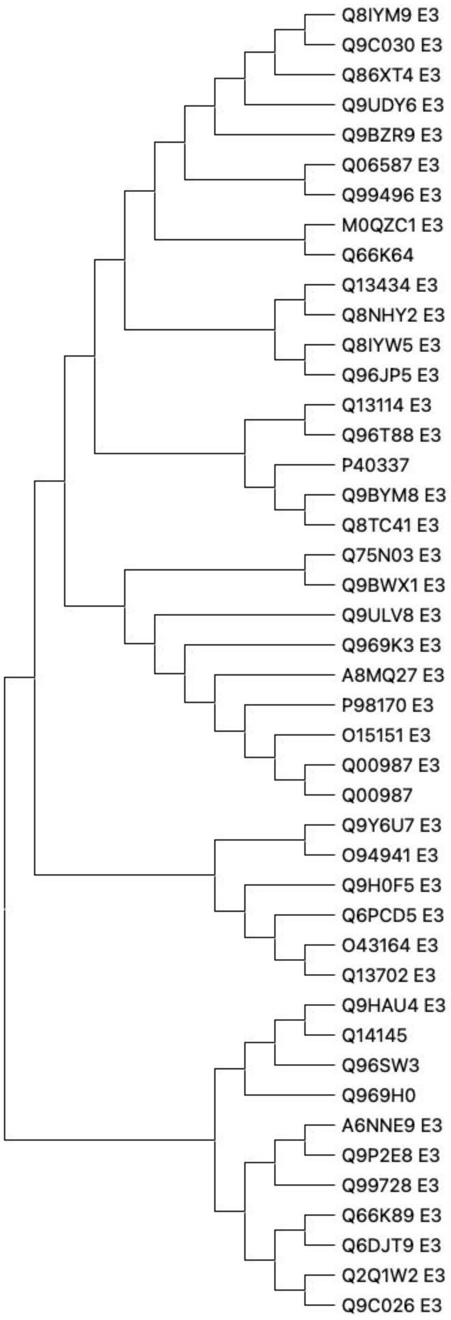

Fig. S3. The phylogenetic tree of the 113 E3 ligases having predicted glues for HER2 along with their relationship to canonical molecular glues.
